# Novel adjunctive technologies for the prevention of colorectal anastomotic leakage: a systematic review

**DOI:** 10.1007/s10151-026-03311-x

**Published:** 2026-03-14

**Authors:** G. Gravante, A. Annicchiarico, R. Melcarne, M. Pollini, L. Vincenti, A. Picciariello

**Affiliations:** 1 Department of General Surgery, Azienda Sanitaria Locale ASL Lecce, Casarano, Italy; 2 Department of Medicine and Surgery, University of Parma, Parma, Italy; 3 Department of Surgery, Azienda Sanitaria Locale Di Parma, Vaio Hospital, Fidenza, Italy; 4 Department of Translational and Precision Medicine, Sapienza University of Rome, Rome, Italy; 5 Department of Experimental Medicine, University of Salento, Lecce, Italy; 6 Surgical Unit, IRCCS de Bellis, Castellana Grotte, 70013 Bari, Italy

**Keywords:** Colorectal surgery, Anastomotic leakage, Anastomotic reinforcement devices, urgical sealants and biomaterials

## Abstract

**Background:**

Anastomotic leakage (AL) remains one of the most serious complications following colorectal surgery, particularly after low anterior resection. In recent years, several adjunctive technologies have been introduced to reinforce anastomotic integrity during the early phases of healing. This systematic review aimed to evaluate the safety, feasibility, and clinical effectiveness of novel mechanical and biological devices designed to prevent AL in colorectal surgery.

**Methods:**

A systematic literature search was conducted in PubMed, Embase, and Scopus. Human studies evaluating innovative adjunctive devices for AL prevention in patients undergoing low anterior resection were included. Studies focusing on transanal tubes, omental wrapping, or suture reinforcement were excluded. The primary outcome was the rate of anastomotic leakage.

**Results:**

Eighteen studies published between 2005 and 2021 met the inclusion criteria. The evaluated technologies were grouped into six categories: bioabsorbable staple-line reinforcements, nitinol-based compression rings, biodegradable intraluminal sheaths, vacuum-anchored diversion systems, fi brin-based sealants, and collagen patches (Seamguard™, C-Seal™, ColonRing™, Colovac™, Tissucol™/Greenplast™, TachoSil™, and Hemopatch™). Considerable heterogeneity in study design and methodological quality was observed. Although several devices demonstrated acceptable technical feasibility and safety, their eff ect on AL prevention was inconsistent. Only a limited number of studies included control groups or were designed as randomized controlled trials. Device-related complications, including migration, technical difficulties, and local tissue reactions, were reported in some series.

**Conclusions:**

Current adjunctive technologies have not consistently demonstrated a reduction in AL rates after colorectal surgery. While selected devices may provide potential benefit in high-risk patients, their clinical applicability remains limited by technical constraints and heterogeneous evidence. Further large, high-quality studies incorporating standardized definitions of AL and patient-specific risk stratification are warranted.

## Introduction

Anastomotic leakage (AL) is among the most serious complications following colorectal surgery [1,2], representing a major cause of postoperative morbidity and mortality [2], having a detrimental impact on both short-term recovery and long-term oncologic outcomes [3,4], and imposing a significant financial burden on hospitals and social care systems [5]. Despite significant improvements in surgical technique and perioperative management, leakage rates remain clinically relevant, particularly in low anterior resections and in patients with specific risk factors [6,7]. Protective ileostomy decreases the incidence of significant clinical effects of AL but does not prevent occurrence [8]. To address this issue, several adjunctive strategies have been developed, including mechanical reinforcements, intraluminal protective devices, and biological sealants, with the aim of enhancing anastomotic integrity and preventing leakage during the critical early healing phase.

A recent umbrella review has comprehensively summarized the evidence from systematic reviews and meta-analyses evaluating traditional protective measures such as transanal decompression tubes (TAT), reinforcing sutures, and omental wrapping [9]. These reviews reported a 43–70% reduction in AL rates with TAT and a 59–75% reduction with reinforcing sutures, particularly among patients without diverting stomas. Moreover, TAT use was associated with a 62–84% decrease in reintervention rates [9]. However, a number of novel technologies with different mechanisms of action have emerged in recent years and are gaining clinical interest. These include bioabsorbable staple line reinforcement materials, nitinol-based compression anastomosis rings, biodegradable protective sheaths, vacuum-anchored intraluminal stents, fibrin-based sealants, and collagen-derived hemostatic patches. Each of these approaches provides a unique protective effect, either by stabilizing the anastomotic line, diverting fecal stream, or promoting tissue sealing, and some have shown promising results in terms of technical feasibility and clinical safety.

The purpose of this systematic review is to provide a comprehensive synthesis of the available evidence on these emerging adjunctive technologies used in colorectal surgery to prevent AL. In doing so, this work specifically excludes interventions that have already been the subject of dedicated reviews, such as TAT, manual suture reinforcement techniques, and omental interposition, to avoid redundancy and focus on devices and materials that represent a distinct and innovative category.

## Materials and methods

The review protocol was registered (registration no. CRD420251101472) a priori in the International Prospective Register of Systematic Reviews (PROSPERO; https://www.crd.york.ac.uk/PROSPERO/view/CRD420251101472). This systematic review was conducted according to the Preferred Reporting Items for Systematic Reviews and Meta-Analyses (PRISMA) guidelines [10,11]. A completed PRISMA 2020 checklist is provided as a Supplementary Table.

Studies were considered eligible if they investigated the use of any mechanical, biological, or physical adjunct aimed at reducing the incidence of AL following colorectal surgery, excluding those focused on TAT, reinforcing sutures, or omental wrapping. Only studies involving human subjects, published in English as full-text articles, and reporting clinically relevant outcomes were included. Considering the limited number of reports present in literature, both prospective and retrospective designs were accepted, including randomized controlled trials, cohort studies, pilot investigations, and case series.

A comprehensive literature search was performed using the databases PubMed, Embase, and Scopus. The search strategy included a combination of keywords and Medical Subject Headings (MeSH) terms related to colorectal anastomosis and specific technologies of interest, including ColonRing, C-Seal, Colovac, Seamguard, fibrin glue, Hemopatch, and TachoSil. The Current Controlled Trials database (www.controlled-trials.com) was also screened to identify ongoing randomized trials. Additionally, the reference lists of relevant articles and prior reviews were screened for further eligible studies. The final search was updated in June 2025, and no restriction on publication year was applied.

Titles and abstracts were screened for relevance, and full texts of potentially eligible studies were independently reviewed by two authors (G.G. and A.A.) in two stages, with any disagreements resolved by discussion or consultation with a third author (A.P.). Data from eligible studies were extracted and compiled into a centralized database. For each selected study, data were extracted regarding study design, country, sample size, patient demographics, type of intervention, surgical indication, surgical approach, AL rate, need for reintervention, presence of device-related complications, and length of hospital stay. Studies were grouped and analyzed according to the specific type of technology investigated, namely compression anastomosis rings, biodegradable intraluminal sheaths, vacuum-anchored diversion devices, bioabsorbable staple line reinforcements, fibrin-based sealants, and collagen patches.

The primary outcome of interest was the rate of AL as reported by the authors. Secondary outcomes, when reported, included other anastomotic complications (i.e., stenosis); the frequency and nature of adverse events related to the device; and the technical success or failure of deployment. Given the heterogeneity of study designs and interventions, a narrative synthesis approach was adopted to describe and compare the findings across studies.

## Results

**Fig. 1:**
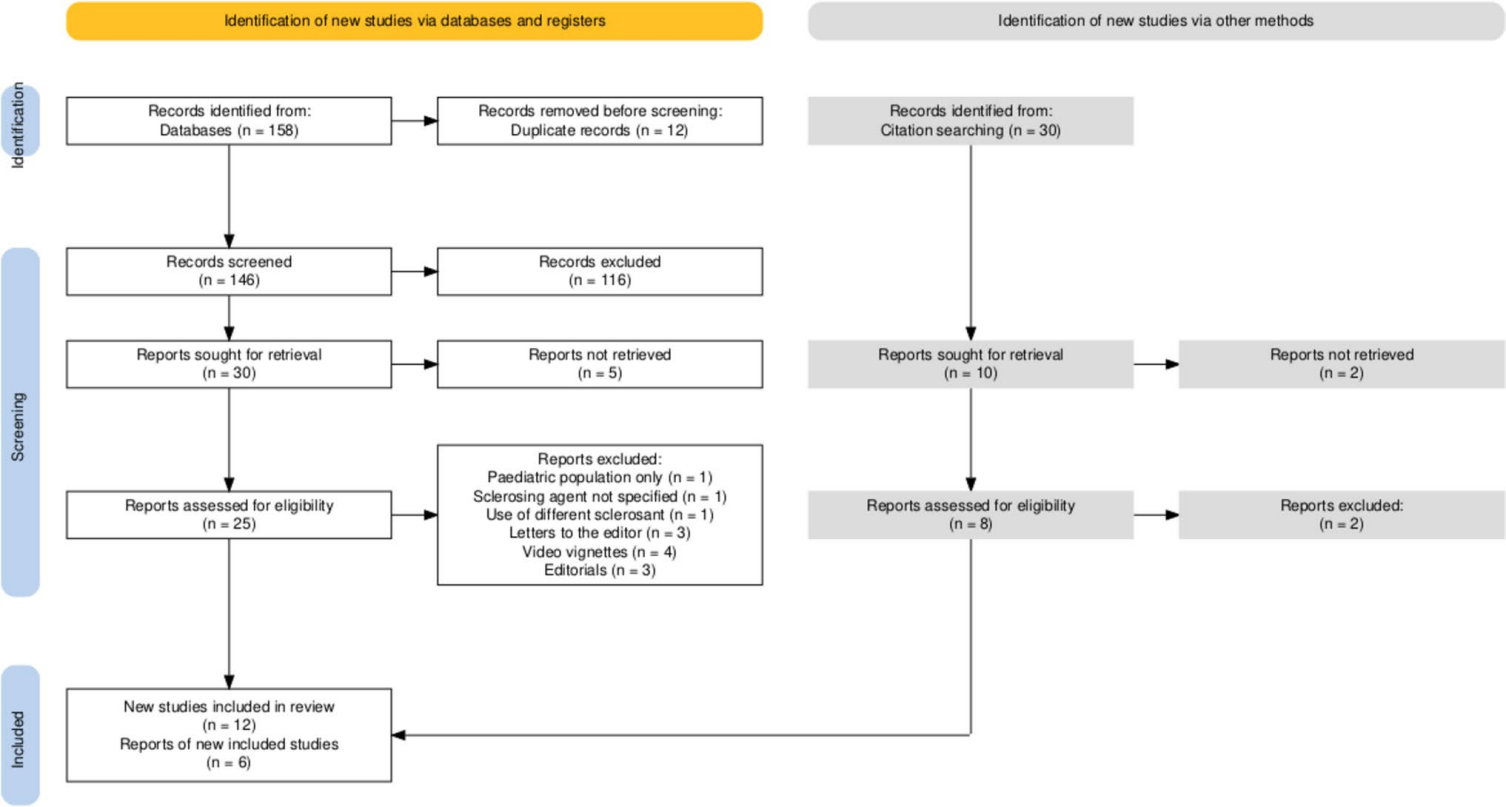
PRISMA flow diagram summarizing the study selection process

A PRISMA flow diagram summarizing the study selection process is shown in Fig. 1. Following title and abstract screening, 18 studies met the eligibility criteria and were included in this systematic review (Table 1) [12–29]. The included reviews were published between 2005 and 2021; 12 studies evaluated mechanical reinforcement devices [12–16,18,19,21–23,25,29], while 6 focused on biological materials [17,20,24,26–28] (Table 1). Among the mechanical devices, the most frequently investigated were the Bioabsorbable Seamguard™ [12,13,21,22,29], and the C-Seal™ [15,16,18]. For biological devices, Tissucol™/Greenplast™ [20,26,27] were the most commonly assessed. Six studies (50%) were conducted in the USA [12,13,22,26,28,29], four were multinational [17,19,23,25], and three originated from the Netherlands [15,16,18] (Table 1). All studies were prospective in design, except for two retrospective analyses [19,20]. Overall, 11 studies adopted a single-arm design [12,13,15–19,23–25,28,29], while 6 included a control group (no reinforcement) [14,20–22,26,27]. On the basis of the Newcastle–Ottawa scale, eight studies were rated as moderate quality [12,13,16–19,25,29], and eight were considered of good to high quality [14,15,20–23,26,27] (Table 2); most studies used validated outcome measures, had sufficient follow-up duration to assess leaks, and reported complete follow-up data for all patients (Table 2). Common limitations included the absence of control groups, small sample sizes, and the resulting inability to perform subgroup analyses (comparisons between risk factors for AL—i.e., distance of the anastomosis from the anal verge, neoadjuvant chemoradiotherapy) (Table 2).

**Table 1:** Overview of study characteristics

Type of device	Device	Authors	Year	Country	Setting	Study design	No. of patients	Sex (percentage [%] male)	Age (years)	Indication	Leakage percentage (%)
Mechanical	Bioabsorbable Seamguard™	Franklin et al. [29]	2005	USA	Single-center	Prospective, single-arm	7^*^	71.4%	76 (46–85)	Mixed	0%
		Franklin et al. [12]	2006	USA	Single-center	Prospective, single-arm	5	40%	56 ± 15	Mixed	0%
		Portillo et al. [13]	2010	USA	Multicenter	Prospective, single-arm	117	47.8%	55 (NR)	Mixed	3.4%
		Placer et al. [21]	2014	Spain	Single-center	RCT	136 versus 145	64.7% versus 62.1%	67 (36–87) versus 66 (33–84)	Mixed	6.6% versus 4.8%^**^
		Senagore et al. [22]	2014	USA	Multicenter	RCT	123 versus 135	48.7% versus 68.9%	54 (18–83) versus 56 (21–82)	Mixed	11.4% versus 12.6%^**^
	C-Seal™	Morks et al. [15]	2010	Netherlands	Single-center	Prospective, single-arm	15	NR	NR	Mixed	0%
		Kolkert et al. [16]	2011	Netherlands	Single-center	Prospective, single-arm	15	73.3%	70 (55–80)	Malignant	0%
		Morks et al. [18]	2013	Netherlands	Multicenter	Prospective, single-arm	37	59%	65 (27–79)	Mixed	3%
	ColonRing™	Tulchinsky et al. [14]	2010	Israel	Multicenter	Prospective, controlled study	23	40% versus 46%	62 ± 12 versus 63 ± 12	Malignant	0% versus 0%^**^
		Masoomi et al. [19]	2013	Multinational	Multicenter	Retrospective, single-arm	1180	43%	64	Mixed	3.2
	Colovac™	D’Urso et al. [23]	2019	Multinational	Multicenter	Prospective, single-arm	15	67%	60 (46–70)	Malignant	26.7%
		De Hous et al. [25]	2023	Multinational	Multicenter	Prospective, single-arm	15	68%	64 (57–71)	Malignant	21.4%
Biological	Tissucol™/Greenplast™	Huh et al. [26]	2010	USA	Single-center	Prospective, controlled study	104 versus 119	58.7% versus 58.0%	62 (25–85) versus 60 (32–82)	Malignant	5.8% versus 10.9%^**^
		Kim et al. [20]	2014	Korea	Single-center	Retrospective, case–control	328 versus 328	63.7% versus 65.8%	69 (40–92) versus 68 (29–94)	Malignant	5.1% versus 7.0%
		Lago-Oliver et al. [27]	2015	Spain	Multicenter	RCT	37	43.3% versus 56.7%	64.0 ± 16.0	Malignant	18.7% versus 52.4%^**^
	TachoSil™	Parker et al. [17]	2012	Multinational	Multicenter	Prospective, single-arm	25	68.0%	65 ± 13	Mixed	8%
	TissuePatch™	Trotter et al. [28]	2018	UK	Single-center	Prospective, single-arm	3^*^	NR	67 (47–70)	Mixed	NR
	Hemopatch™	Kornfeld et al. [24]	2021	Sweden	Single-center	Prospective, single-arm	10	70%	68 (50–94)	Malignant	10%

^*^Patients undergoing low anterior resections out of the total reported, for which specific data were available in the results section

^**^Experimental versus control group

*RCT* randomized controlled trial, *NR* not reported

**Table 2:**
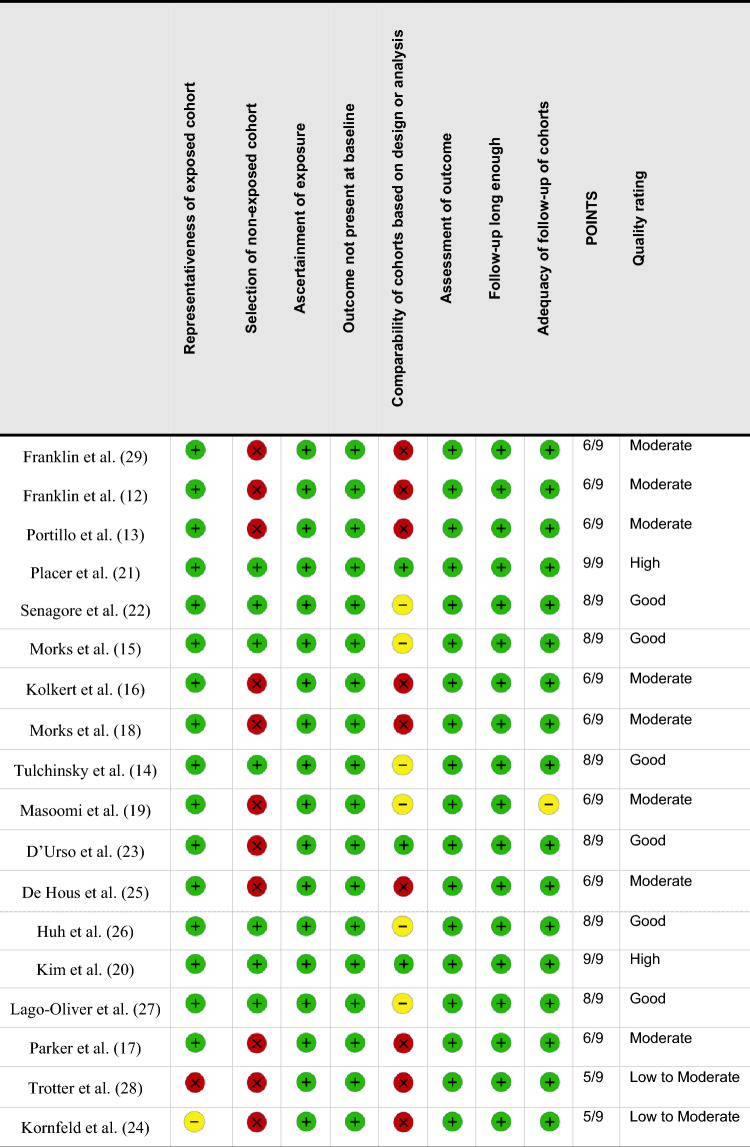
Newcastle–Ottawa scale of included studies

### Mechanical devices

#### Bioabsorbable seamguard™

Bioabsorbable Seamguard™ (W.L. Gore and associates, Flagstaff, AZ, USA) is a porous, bioresorbable reinforcement material made from a synthetic copolymer of glycolide and trimethylene carbonate [30]. Biodegraded through both hydrolytic and enzymatic pathways, the material has demonstrated excellent biocompatibility and is widely used in absorbable sutures and surgical implants. When applied to linear or circular staplers, it is secured with a braided polyester suture and fully absorbed within 6 months. While its use is well established in bariatric surgery, its role in colorectal anastomosis remains less defined [31,32].

Initial clinical applications focused on reinforcing the rectal stump, as described by Franklin et al. in 2005, with further reports in 2006 documenting five successful colorectal anastomoses without leaks in patients with both benign and malignant disease [29,33]. A larger prospective multicenter study by Portillo et al. in 2010 included 117 patients undergoing various left-sided colorectal resections. The leak rate was low (approximately 4%), and although no device-related complications were reported, 18 cases of tearing or displacement necessitated additional Seamguard™ loads (Table 3) [13]. Subsequent randomized controlled trials yielded mixed results. Placer et al. found no significant difference in leak rates between patients treated with Seamguard™ (*n* = 136) and controls (*n* = 145), though a nonsignificant trend toward fewer strictures was observed in the Seamguard™ group (four versus ten cases) [21]. Conversely, Senagore et al. demonstrated a significantly lower incidence of anastomotic stenosis (1 versus 11; *p* = 0.006) in patients receiving reinforcement, despite no difference in leak rates (14 versus 17; *p* = 0.85) [22].

**Table 3:** Device-related adverse events (anastomotic leaks excluded)

Type of device	Device	Authors	Overall frequency of adverse events *n* (%)	Type of adverse events
Mechanical	Bioabsorbable Seamguard™	Franklin et al. [29]	0%	None
		Franklin et al. [12]	0%	None
		Portillo et al. [13]	0%	None
		Placer et al. [21]	–	–
		Senagore et al. [22]	–	–
	C-Seal™	Morks et al. [15]	–	–
		Kolkert et al. [16]	2 (13.3%)	Sheath detachment from the anvil of the stapler during its withdrawal
		Morks et al. [18]	2 (5%)	Inability to extract the seal C after firing: transanal section necessary
	ColonRing™	Tulchinsky et al. [14]	4 (17.4%)	Excessive strength to operate the Colo-ing™ (*n* = 1); non-spontaneous expulsion of the ring (*n* = 2); discomfort from detached ring in the rectal lumen (*n* = 1)
		Masoomi et al. [19]	5 (0.42%)	Non-spontaneous expulsion of the ring (*n* = 1); misfiring (*n* = 1); ‘‘doughnuts’’ not intact: re-anastomosis (*n* = 3)
	Colovac™	D’Urso et al. [23]	8 (53.3%)	Accidental extraction of the Colovac during removal of the introducer (*n* = 1); migration before the end of the implantation period (*n* = 3); minor bleeding (*n* = 4)
		De Hous et al. [25]	2 (13%)	Migration before the end of the implantation period (*n* = 2)
Biological	Tissucol™/Greenplast™	Huh et al. [26]	–	–
		Kim et al. [20]	–	–
		Lago-Oliver et al. [27]	–	–
	TachoSil™	Parker et al. [17]	0%	None
	TissuePatch™	Trotter et al. [28]	–	–
	Hemopatch™	Kornfeld et al. [24]	–	–

“–” Not reported

#### C-Seal device

The C-Seal is a biodegradable polyurethane sheath designed to protect colorectal anastomoses by diverting fecal flow during early healing [15,16,18]. It is glued to the circular stapler anvil before insertion and removed transanally after firing [16].

An initial pilot study by Morks et al. in 15 patients undergoing rectal resection reported successful application of the C-Seal without clinical or subclinical anastomotic leaks (AL), indicating safety and efficacy [15]. Building on this, a 2013 prospective phase II study with 37 patients assessed AL requiring reintervention within 3 months postoperatively. While about half had diverting stomas, which may have lessened AL severity, the C-Seal was applied successfully in 95% of cases. Only one patient (3%) developed AL, requiring surgery; secondary complications included a few perianastomotic abscesses, one rectovaginal fistula managed conservatively, and radiologic leaks without clinical consequence. Surgeon feedback was largely positive [18].

In a separate series of 15 patients, the C-Seal proved compatible with standard staplers and technically straightforward [16]. Although gluing the device to the stapler anvil was slightly inconvenient, it added less than 10 min to the procedure. No major anastomotic complications occurred, and all staple donuts were intact. Two cases involved sheath detachment during withdrawal, requiring manual transanal retrieval but causing no major issues (Table 3). Patients experienced minimal loose stool incontinence while the sheath was in place. The original material degraded within 5 days, prompting a modified version that lasted a median of 13 days in the bowel. One patient experienced prolonged ileus necessitating re-laparotomy unrelated to the C-Seal, and the median hospital stay was 8 days (range 7–53) [16].

#### ColonRing™

The ColonRing™ (NiTi Shape Memory BioDynamix ColonRing™) is a Food and Drug Administration (FDA)-approved, nickel–titanium (NiTi) compression anastomosis device designed for end-to-end colorectal anastomoses in both open and laparoscopic surgery [34–36]. Made of nitinol, a shape-memory alloy, the ring applies continuous, uniform pressure regardless of tissue thickness and facilitates sutureless, hemostatic anastomoses without leaving permanent foreign material [14,19,37–40]. The system includes a plastic anvil and a nitinol-based base ring that compresses the bowel ends after activation. The compressed tissue undergoes necrosis, and the ring is naturally expelled within 8–10 days [39,40].

A first-in-human study by Tulchinschy et al. found the device comparable in safety and efficacy to conventional stapling, with no anastomotic leaks and only minor complications unrelated to the technique. In two patients, the ring required manual removal (Table 3), but healing and recovery were otherwise uneventful [14]. In a large retrospective cohort of 1180 patients, the ColonRing™ showed a low leak rate (3.22%) and consistent performance across laparoscopic and open procedures [19]. The device was well tolerated, with a median expulsion time of 8 days and minimal technical complications (Table 3). No perioperative deaths were reported, and the system was considered intuitive and easy to integrate into standard practice [19].

#### Colovac device

The Colovac device (Colovac, SafeHeal, France) is a single-use intraluminal bypass system designed to protect low colorectal anastomoses by diverting fecal flow during the early postoperative period. It consists of a flexible polymer sheath attached to a vacuum-anchored stent, deployed 20 cm above the anastomosis and protruding about 5 cm from the anus for monitoring. The vacuum system secures mucosal apposition and stabilizes the device in place for up to 14 days [23,25].

In a first-in-human trial with 15 patients, device placement was complication-free, with a median insertion time of 7 min and 93% of procedures rated as easy [23]. Endoscopic removal was successful in 14 cases, with no fecal passage detected below the anchoring site. Anastomotic protection was achieved in 80% of patients, avoiding stoma creation in 67%. However, 27% developed leaks, mostly linked to device migration (Table 3), which occurred prematurely in 20% of cases owing to vacuum failure or misplacement. Despite these issues, patient tolerance was generally good [23].

Further support comes from the SAFE and SAFE-2 studies, which evaluate Colovac and its upgraded version, Colovac+ , for temporary fecal diversion after low anterior resection [25,41]. In the SAFE study (*n* = 15), Colovac+ implantation was successful in all patients, with 87% rating the procedure as easy or very easy. The device allowed stoma avoidance in 73% of cases and achieved effective fecal diversion throughout the 10-day implantation. Four patients (27%) developed anastomotic defects, none directly attributed to the device, and only 13% experienced clinically relevant migration (Table 3) [25]. The ongoing SAFE-2 trial is a large international RCT enrolling 342 patients to assess Colovac’s safety and efficacy in reducing diverting stoma rates. While final results are pending, preliminary data confirm the device’s feasibility and potential benefits [41]; nonetheless, AL and device migration remain important challenges.

### Biological devices

#### Tissucol™ and Greenplast™

Fibrin glues (Tissucol™—Baxter, Vienna, Austria; and Greenplast™—Green Cross Corporation, Yongin, Korea) have been widely used across various surgical fields to reduce lymphorrhea and serous effusions, control bleeding, prevent biliary leaks after liver resections, and secure meshes in hernia repairs [42–45]. Some studies in upper gastrointestinal surgery suggest fibrin glue’s role extends beyond mechanical sealing to potentially enhancing healing and improving suture outcomes [46]. In colorectal surgery, fibrin glue is considered a tool to reduce anastomotic leaks, though animal studies show mixed results [47–51]. It has also been proposed that fibrin glue might mechanically inhibit tumor cell growth and spread at the anastomosis site [52].

The first clinical experience was reported by Hu et al. in 2010, who compared laparoscopic colorectal resections in patients treated with fibrin glue (Tissucol™ and Greenplast™) with a control group. They found no significant difference in anastomotic leak rates between the two groups (*p* = 0.169) [26]. In a larger study, Kim et al. analyzed over a thousand patients with rectal cancer and identified absence of Tissucol™/Greenplast™, advanced tumor stage, and low tumor location as independent risk factors for leakage. However, when comparing matched groups, fibrin glue use did not significantly reduce leak rates [20]. Conversely, a smaller randomized trial by Lago-Oliver et al. in 2015 demonstrated that applying Tissucol™ or Greenplast™ around the anastomosis significantly lowered both leaks and reoperations, although it had no effect on mortality [27].

#### Tachosil™

TachoSil™ (Takeda Pharmaceuticals, Zurich, Switzerland) is a fibrinogen- and thrombin-coated equine collagen sponge used as a hemostatic and sealing adjunct in various surgical procedures. Upon contact with blood or physiological fluids, the active coating dissolves and forms a fibrin clot that adheres the matrix to tissue surfaces, promoting both hemostasis and sealing through a stable fibrin mesh [53]. Its efficacy has been demonstrated in vascular and pancreatic surgery [54], and preclinical studies suggest it may also enhance healing in colonic anastomoses [55,56].

In a prospective, nonrandomized multicenter study, Parker et al. assessed the feasibility of TachoSil™ application to colorectal anastomoses in 25 patients, 80% of whom had cancer [17]. Application was considered feasible in 63% of evaluable cases, though technical limitations prevented full assessment in several patients. Most required two patches to achieve adequate coverage. Ten major adverse events occurred, including two anastomotic leaks. Despite this, the authors concluded that TachoSil™ was safe and technically applicable, although the study did not demonstrate a definitive benefit in leak prevention [17].

#### TissuePatch™

TissuePatch™ (Tissuemed, Leeds, UK) is a sterile, resorbable, self-adhesive surgical sealant composed of four layers of poly(lactide-co-glycolide) and poly(*N*-vinylpyrrolidone-co-acrylic acid-co–*N*-hydroxysuccinimide ester). These layers provide structural support and an antiadhesion barrier. Upon application, it bonds instantly to tissue through ionic and covalent interactions, preventing fluid or air leaks, and is gradually absorbed. Widely used in thoracic, head and neck, and neurosurgery, it reduces dural and air leaks [57]. However, its use in gastrointestinal anastomoses is limited. Trotter et al. applied TissuePatch™ around colorectal anastomoses in nine patients, but the study was stopped early owing to high morbidity: Six patients had major complications, including leaks and abscesses [28]. The authors suggested the patch may hinder healing by creating a detrimental microenvironment [28].

#### Hemopatch™

Hemopatch™ (Baxter AG, Vienna, Austria) is an absorbable, collagen-based hemostatic patch coated with polyethylene glycol (PEG). It is designed to control bleeding and seal leaks of fluids or air when conventional methods are insufficient, and can also be used to close dural defects caused by trauma or surgery [58,59].

In 2021, Kornfeld evaluated the feasibility of Hemopatch™ in a case series of ten patients undergoing rectal resection with anastomoses located more than 10 cm from the anal verge [24]. The aim was to cover 75% of the anastomotic circumference, focusing on the posterior area, which is most prone to leaks. Application difficulty varied, being easy in two cases, moderately difficult in five, and very difficult in one, while in two cases, the patch was not applied per surgeon decision. Although only one patient developed a leak and conclusions are limited, the author found Hemopatch™ application to be a reproducible and effective technique [24].

## Discussions

The pursuit of strategies to reduce AL in colorectal surgery has led to the development of a variety of mechanical and biological reinforcement devices, each aiming to improve local conditions at the anastomotic site. While initial enthusiasm was fueled by promising experimental data and early clinical feasibility studies [13,19,29,33], the overall clinical evidence remains inconsistent [21–23,25], with limited high-quality trials and heterogeneous results (Table 1). Several mechanical devices aim to provide structural reinforcement at the time of anastomosis construction. The Bioabsorbable Seamguard™, for instance, is one of the most widely studied, but despite good biocompatibility and extensive use in bariatric surgery [60,61], its efficacy in colorectal procedures is far from established. Over the years, studies have progressively improved the scientific quality (Table 1), but recent randomized trials have failed to consistently demonstrate reductions in AL [21,22]. Similarly, compression-based systems such as the ColonRing™ offer an elegant, sutureless approach that avoids permanent foreign materials, but despite encouraging retrospective data on 1180 patients [19], robust prospective evidence is still lacking [14]. Some devices, such as the C-Seal and Colovac, represent a conceptual shift by attempting to modulate the early intraluminal environment. By temporarily diverting fecal flow away from the anastomosis, these systems aim to minimize contamination during the critical healing window [15,16,18,23,25]. Early phase trials have not only demonstrated technical feasibility and acceptable tolerance [15,23] but also highlighted device-specific challenges: migration, incomplete sealing, or technical complexity (Table 3) [23,25]. Moreover, the relatively high rates of AL seen in some Colovac studies, albeit not always clinically significant, raise concerns about the reliability of protection [23,25]. Overall, findings of mechanical devices reflect a broader challenge: Even sophisticated mechanical barriers cannot fully compensate for suboptimal surgical conditions or high-risk patient profiles.

Biological adjuncts offer a different logic—rather than excluding fecal content or reinforcing anastomotic staples, they aim to interact with tissue biochemistry to promote healing. Fibrin glues such as Tissucol™ and Greenplast™ have been used for decades in other surgical fields [42–45], and their application to colorectal anastomoses appears biologically plausible. Yet clinical trials have shown mixed results, some reporting reduced leak rates [27] and others failing to show significant differences [26]. Large cohort studies initially suggested an association between glue absence and leak, but these findings were confounded by surgical heterogeneity and absence of control for confounding factors [20]. Furthermore, the proposed antitumor effect of fibrin glues at the anastomotic site remains speculative and unsupported by robust clinical data [52]. More recently, collagen-based matrices such as TachoSil™ and Hemopatch™ have been evaluated [17,24]. These devices are appealing for their dual action—hemostatic and sealing—but their practical deployment around colorectal anastomoses is not without difficulties. In deep pelvic fields or with bulky mesentery, achieving adequate coverage may be technically challenging, and their contribution to leak prevention remains unproven [17,24]. Perhaps more concerning are results from the application of TissuePatch™, which was associated with high complication rates in a small series and raises questions about the potential of certain materials to impair, rather than support, healing [28].

This review is subject to several limitations that should be acknowledged. First, many of the included studies did not clearly differentiate between high, mid, and low colorectal anastomoses, despite the well-established variability in leak risk based on anatomical level. This lack of stratification—evident in studies such as those by Trotter et al. [28], Lago-Oliver et al. [27], and Portillo et al. [13]—limits the interpretability and generalizability of the reported outcomes. Second, protective ileostomies were used in a subset of patients in several studies, yet outcomes were often reported without separating stoma and non-stoma groups. This introduces a potential confounder, as diversion may independently reduce the clinical impact of anastomotic leaks and bias the perceived effectiveness of reinforcement devices. Third, preoperative neoadjuvant chemoradiation, a known risk factor for impaired anastomotic healing, was present in some patient populations—for example, in the study by Huh et al. [26]—but was not consistently accounted for in subgroup analyses. In addition, small sample sizes and inconsistent AL definitions further increased the heterogeneity in baseline risk, complicating the interpretation of comparative outcomes and precluding any reliable meta-analysis. Finally, and equally important, many studies included in this review were sponsored or supported by device manufacturers, thus introducing potential biases associated with industry-funded trials.

## Conclusions

Anastomotic healing is a complex, multifactorial process, and no device—whether mechanical or biological—has shown the ability to consistently overcome the vulnerability of the colorectal anastomosis, particularly in low rectal resections. Mechanical or biological reinforcements may offer marginal improvements, especially in selected high-risk patients, but cannot replace meticulous surgical technique, careful patient selection, and optimal perioperative management. Moreover, technical issues such as application time, device fixation, and anatomical constraints often limit the real-world utility of these technologies. The use of reinforcement devices should be applied within clinical trials or in selected cases where conventional protection strategies—such as diverting stomas—are not feasible (i.e., cultural or patient resistance).

## Data Availability

No datasets were generated or analyzed during the current study.
